# Benchmarking Barren Plateau Mitigation Strategies in Quantum Neural Networks on Standard and Medical Image Datasets

**DOI:** 10.3390/jimaging12070275

**Published:** 2026-06-23

**Authors:** Maqsudur Rahman, Rui Liu, Anup Majumder, Pintu Chandra Paul, Kangtong Mo, Amena Begum, Kashmi Sultana, Nahida Akter, Lu Wei, Ye Zhang, Jun Zhuang

**Affiliations:** 1 Department of Computer Science, Boise State University, Boise, ID 83706, USA; 2 Department of Information and Communication Technology, Comilla University, Cumilla 3500, Bangladesh; 3 College of Computing, Illinois Institute of Technology, Chicago, IL 60616, USA; 4 Department of Computer Science and Engineering, Jahangirnagar University, Savar 1342, Bangladesh; 5 The Grainger College of Engineering, University of Illinois Urbana-Champaign, Champaign, IL 61820, USA; 6 Data Science Department, Stony Brook University, Stony Brook, NY 11794, USA; 7 Independent Researcher, Portland, OR 97217, USA

**Keywords:** barren plateaus, quantum neural networks, quantum machine learning, benchmark, trainability, initialization strategies, medical image datasets

## Abstract

Barren plateaus (BPs) pose a major trainability challenge for quantum neural networks (QNNs) by causing gradients to concentrate near zero as circuit size, depth, or expressibility increases. This study presents a comparative benchmark of 10 BP mitigation strategies across six qubit settings (2, 4, 8, 12, 16, and 20) and three datasets of increasing complexity: Iris, MNIST, and MedMNIST. The evaluated methods include eight initialization-based strategies (Beta, Gaussian, Uniform Norm, CNN-based initialization, He-normal, He-uniform, Xavier-normal, and Xavier-uniform), one model-based variational encoder, and one optimization-based time-nonlocal Fourier parameterization. Experiments were implemented using PennyLane 3.10 and PyTorch 2.5 with simulator backends. We evaluate trainability using gradient variance and training loss, and we clarify that the benchmark analyzes simulated QNN optimization behavior rather than hardware-noise-resilient or noisy-label learning. Across the tested two-layer circuit configurations, the mitigation strategies maintained measurable gradient variance and stable loss reduction, suggesting that severe barren plateau behavior was not observed under the benchmark conditions. CNN-based and Beta initialization showed strong empirical behavior in variance retention and convergence speed, while Gaussian initialization was comparatively weaker in higher-dimensional settings. The study provides a reproducible benchmark structure for comparing BP mitigation behavior and identifies important limitations related to circuit depth, hardware noise, feature encoding, and classification performance that should be addressed in future QNN benchmarking.

## 1. Introduction

The NISQ era marks a transformative period in quantum computing, where quantum devices with limited qubits and inherent noise provide both opportunities and challenges for advancing computational capabilities [1,2]. Despite their limitations, these devices have facilitated the development of hybrid quantum–classical algorithms, particularly Variational Quantum Algorithms (VQAs) [3]. VQAs leverage parameterized quantum circuits (PQCs) optimized through classical methods to solve complex problems in machine learning, quantum chemistry, and optimization [4,5].

In medical imaging, robustness is also important because annotations may be uncertain or inconsistent; related classical studies have examined unreliable annotations in segmentation and diagnosis workflows [6]. Although the present benchmark does not model noisy labels, this broader context motivates future extensions that connect QNN trainability with medical-image robustness. Quantum Neural Networks (QNNs), a subset of VQAs, have emerged as powerful tools for leveraging quantum mechanics to address computationally intensive tasks [7,8]. QNNs combine the expressibility of PQCs with quantum-enhanced optimization techniques, enabling applications in fields like supervised learning [9,10], molecular simulation [11], and combinatorial optimization [12]. By exploiting quantum parallelism, QNNs hold the potential to outperform classical neural networks in scenarios involving high-dimensional data and entangled correlations [13].

However, the scalability of QNNs and VQAs is hindered by a critical bottleneck: the barren plateau (BP) phenomenon. BPs refer to regions in the parameter space where the gradient of the cost function vanishes exponentially as system size increases, resulting in flat optimization landscapes that impede gradient-based training [14]. This challenge becomes particularly acute in deep circuits and high-dimensional parameter spaces, making BP mitigation a cornerstone of ongoing research in quantum machine learning. Addressing the BP phenomenon is essential for the development of scalable, trainable QNNs capable of solving real-world problems in the NISQ era [15].

### 1.1. Motivation and Objectives

Barren plateaus remain a central obstacle to scaling quantum neural networks because vanishing gradients can make gradient-based optimization ineffective. Although many mitigation strategies have been proposed, they are often evaluated under different circuits, datasets, qubit counts, optimizers, and reporting conventions, making direct comparison difficult. A controlled benchmark is therefore useful for understanding how different mitigation families behave under shared experimental conditions.

The objective of this study is to compare initialization-based, model-based, and optimization-based BP mitigation strategies using a common QNN architecture, optimizer, training length, and set of qubit configurations. The benchmark focuses on optimization behavior, measured primarily through gradient variance and training loss, across Iris, MNIST, and MedMNIST. Because the experiments use simulator backends, the study should be interpreted as a controlled trainability benchmark rather than as a demonstration of robustness to real quantum hardware noise.

The study has three main goals. First, it establishes a reproducible benchmark for comparing BP mitigation strategies across multiple qubit configurations and datasets. Second, it analyzes whether the evaluated strategies maintain measurable gradient variance and stable loss reduction under the tested conditions. Third, it provides an open implementation framework that can be extended to deeper circuits, physical quantum hardware, additional medical datasets, and explicit noise models.

### 1.2. Contributions

This paper makes the following contributions. First, it provides a comparative benchmark of 10 BP mitigation strategies, including eight initialization-based methods, one model-based variational encoder, and one optimization-based time-nonlocal Fourier parameterization, evaluated under the same QNN training setup. Second, it compares these strategies across six qubit settings and three datasets, enabling an empirical view of how trainability behavior changes with input complexity and qubit count. Third, it analyzes gradient variance and training loss jointly, allowing the benchmark to distinguish between gradient retention and practical convergence behavior. Fourth, it documents important methodological limitations, including the use of simulator backends, a two-layer circuit design, and the absence of explicit hardware noise models, thereby defining a clear path for future hardware-aware and noise-aware BP benchmarking.

### 1.3. Paper Organization

The remainder of this paper is organized as follows. Section 2 reviews the barren plateau phenomenon and the main factors that influence QNN trainability. Section 3 describes the datasets, preprocessing, quantum feature encoding, QNN setup, mitigation strategies, and evaluation metrics. Section 4 presents the variance and loss analyses and discusses circuit-depth, scalability, and practical QNN limitations. Section 5 summarizes related work on initialization-based, model-based, optimization-based, and diagnostic approaches for BP mitigation. Section 6 concludes the paper and outlines limitations and future research directions.

## 2. Preliminary Background

The barren plateau (BP) phenomenon poses a significant challenge in training parameterized quantum circuits (PQCs) and quantum neural networks (QNNs), which are central to quantum machine learning (QML) and variational quantum algorithms (VQAs). Barren plateaus are characterized by exceedingly flat optimization landscapes, where the gradients of the cost function diminish exponentially with the problem size. This renders gradient-based optimization methods ineffective, creating a critical bottleneck for advancing quantum machine learning applications.

### 2.1. Parameterized Quantum Circuits (PQCs) and Cost Function Landscape

Parameterized quantum circuits are the computational backbone of QML. They utilize tunable parameters $\theta =(\theta_{1},\theta_{2},\ldots ,\theta_{m})$ to control quantum gates that manipulate the quantum state |ψ(θ)〉. A typical PQC begins with a simple quantum state, such as ${|0\rangle }^{⊗n}$, and sequentially applies parameterized quantum gates, such as rotation gates in Equation (1)

(1) $$R_{X}\left(\theta \right)=e^{-i\theta X/2},$$

interleaved with entangling gates like controlled-*Z* (CZ) gates.

The circuit’s performance is quantified by measuring the expectation value of an observable *O*, defining the cost function C(θ) as follows (Equation (2))

(2) C(θ)=〈ψ(θ)|O|ψ(θ)〉.

The objective is to optimize the parameters θ to minimize or maximize C(θ) using classical gradient-based optimization methods.

### 2.2. Manifestation of Barren Plateaus

Barren plateaus emerge when gradients concentrate near zero over large regions of the parameter space, often causing the cost-function landscape to appear nearly flat to gradient-based optimizers. This flatness, signified by negligible gradients, results in the following core challenges.

#### 2.2.1. Vanishing Gradients

The variance of the gradient $\frac{\partial C}{\partial \theta }$ diminishes exponentially with the number of qubits as *n* in Equation (3)

(3) $$\mathrm{Var}\left(\frac{\partial C}{\partial \theta }\right)\propto \exp\left(-\alpha n\right),$$

where α>0 is a constant determined by the circuit depth and architecture. This exponential decay makes it increasingly difficult to locate optimal parameters as the system size grows, especially in high-dimensional quantum systems.

#### 2.2.2. Flat Optimization Landscapes

In barren plateaus, the cost function exhibits minimal sensitivity to parameter updates such as in Equation (4)

(4) C(θ+δθ)≈C(θ),

for small parameter shifts δθ. This indicates that the gradients are insufficiently large to guide the optimization process, causing classical optimizers to stagnate.

### 2.3. Relevance to Quantum Neural Networks

Barren plateaus fundamentally limit the scalability of QNN algorithms, particularly those reliant on variational quantum circuits. As the number of qubits increases, the likelihood of encountering barren plateaus rises, necessitating careful circuit design, initialization strategies, and optimization techniques to mitigate this phenomenon. Addressing barren plateaus is vital for enabling PQCs to unlock their full potential in solving practical problems across fields such as quantum chemistry, machine learning, and optimization.

### 2.4. Theoretical Foundations and Indicators

Barren plateaus are closely linked to the structure of PQCs, including their depth, entanglement patterns, and parameter initialization. Recent theoretical studies have highlighted the dependence of barren plateaus on circuit design and problem structure, emphasizing the role of gradient variance as a key metric to diagnose and mitigate these challenges. By systematically evaluating the variance of gradients across the parameter space, researchers can identify conditions under which barren plateaus are likely to occur and develop targeted strategies to overcome them.

This understanding of barren plateaus serves as a foundation for the methodologies and evaluation metrics employed in this study, ensuring that the quantum circuits and optimization strategies are designed to minimize the impact of flat landscapes, thereby enabling efficient training of quantum neural networks.

### 2.5. Key Factors Contributing to Barren Plateaus

1 **Expressibility of Parameterized Quantum Circuits (PQCs)**: Highly expressive PQCs, which can approximate arbitrary unitary transformations, tend to create more entanglement among qubits. While expressibility is a desirable feature for capturing complex correlations, it also increases the likelihood of gradients vanishing. The variance of gradients decreases as circuits become more expressive, as shown by the relationship in Equation (5)
  (5) $$\mathrm{Var}\left(\frac{\partial C}{\partial \theta }\right)=\frac{\mathrm{Tr}\left(\rho^{2}\right)-\mathrm{Tr}\left(\sigma^{2}\right)}{\dim(H)},$$
  where ρ and σ are density matrices, and dim(H) represents the Hilbert space dimension [16].
2 **Entanglement-Induced BPs**: Marrero et al. demonstrated that BPs can also arise from excessive entanglement in the circuit. Deeply entangled states lead to near-uniform sampling in the Hilbert space, which further diminishes gradient variance. This effect is exacerbated in circuits with global cost functions, as they involve measurements over all qubits, amplifying the gradient vanishing effect [17].
3 **Cost Function Locality**: Cerezo et al. showed that the structure of the cost function significantly impacts the emergence of BPs. Local cost functions, which involve a small subset of qubits, tend to mitigate gradient decay compared to global cost functions. This is formalized as follows in Equation (6)
  (6) $$\mathrm{Var}\left(\frac{\partial C}{\partial \theta }\right)∼\frac{1}{\mathrm{poly}(n)}.$$
  where the polynomial factor depends on the locality of the cost function [14].
4 **Initialization and Parameter Scaling**: The choice of parameter initialization plays a crucial role in determining the onset of BPs. Improper initialization can cause the circuit to operate in regions of parameter space where gradients are uniformly small. Techniques like Beta Initialization and Fourier-based parameterization have been proposed to address this issue by ensuring the variance of initial parameters is distributed optimally across the circuit layers [17].

## 3. Methodology

This study systematically evaluates multiple barren plateau (BP) mitigation techniques within quantum neural networks (QNNs), employing initialization-based, model-based, and optimization-based strategies. Each approach targets different aspects of the training process to improve gradient flow, stability, and scalability, particularly in complex datasets. The following sections describe the datasets, experimental setup, BP mitigation techniques, and evaluation metrics used in this research.

### 3.1. Scope of Noise Modeling

The experiments in this study were conducted using PennyLane simulator backends and do not explicitly model hardware noise, open-system decoherence, imperfect gate fidelities, readout error, or noisy labels. Therefore, the benchmark evaluates BP mitigation behavior in controlled simulated QNN training rather than noise-resilient performance on physical NISQ devices. The phrase NISQ is used as motivation for the relevance of trainability and scalability, but the reported results should not be interpreted as hardware-noise validation.

### 3.2. Datasets

To assess the effectiveness and robustness of BP mitigation strategies, we selected three datasets representing a range of complexities:

1 **Iris**: A small dataset with 150 samples and four features across three classes, commonly used for evaluating the basic performance of classification algorithms. It serves as a benchmark for assessing BP mitigation in simpler QNN configurations.
2 **MNIST**: A dataset containing 70,000 grayscale images of handwritten digits (0–9), each 28 × 28 pixels. This dataset offers moderate complexity and is frequently used to evaluate model performance in classification tasks, including QNNs.
3 **MedMNIST**: A high-dimensional dataset composed of medical images from various categories, with greater complexity and dimensionality. This dataset is ideal for evaluating BP mitigation strategies in large-scale QNN applications, testing scalability and the ability to handle intricate data structures.

### 3.3. Preprocessing and Quantum Feature Encoding

All datasets were normalized before being passed to the QNN pipeline. Iris samples were used as tabular feature vectors. For MNIST and MedMNIST, image samples were converted into vector representations and then reduced to match the number of input features required by each qubit configuration. Specifically, an *n*-qubit experiment used an *n*-dimensional feature vector, where n∈{2,4,8,12,16,20}. The reduced feature vector was encoded into the quantum circuit using rotation-based feature encoding, so each selected feature controlled a parameterized rotation gate.

In this study, MedMNIST is used as the representative medical-image benchmark source in the implemented pipeline, rather than as a comparative study across individual MedMNISTv2 subsets. MedMNISTv2 provides multiple biomedical image datasets; however, the present work uses the MedMNIST configuration implemented in the benchmark code as a general medical-image setting for evaluating QNN trainability. The purpose of including MedMNIST is to test barren-plateau mitigation behavior on medical image data with greater visual and structural complexity than Iris and MNIST, not to draw disease-specific conclusions or compare performance across MedMNISTv2 subsets.

This preprocessing step is important because raw image dimensions are much larger than the number of available qubits in near-term QNN experiments. Therefore, dimensionality reduction or feature selection is necessary before quantum encoding. In the current benchmark, feature encoding is treated as a controlled preprocessing step rather than as a separate optimization target. Future work should compare alternative encodings, such as angle encoding, amplitude encoding, and patch-based encodings, because these choices differ substantially in circuit depth, state-preparation cost, qubit requirements, and scalability.

### 3.4. Experimental Setup

This section provides a comprehensive overview of the environment setup, including the configurations for quantum neural networks (QNNs) and hyperparameters with necessary computational settings used in this study. The setup ensures reproducibility and facilitates rigorous benchmarking across diverse methods and datasets.

#### 3.4.1. Quantum Neural Network Setup

The QNNs in this study are implemented using PennyLane (version 0.30.0) and PyTorch (version 2.0.1) frameworks. PennyLane’s default.qubit simulator is employed for CPU-based simulations, while lightning.qubit is used for GPU acceleration. The QNN consists of parameterized quantum circuits (PQCs) with the following configurations:

- **Number of qubits:** Experiments are conducted for 2, 4, 8, 12, 16, and 20 qubits.
- **Circuit layers:** Each circuit includes two parameterized layers with single-qubit rotations (RX, RY) and controlled-Z (CZ) entangling gates.
- **Measurement:** The expectation value of the Pauli-Z observable is computed for the final state.

#### 3.4.2. Hyperparameters

The following hyperparameters are optimized to ensure fair comparisons:

- **Learning rate:** 0.001 (scaled for specific initialization strategies when required).
- **Batch size:** 16 samples per batch for all datasets.
- **Optimizer:** Adam optimizer is employed for all experiments, with adaptive learning based on initialization.
- **Epochs:** Each experiment is run for 30 epochs.

### 3.5. BP Mitigation Techniques

- **Initialization-Based Strategy**
  1 **Beta Initialization**: Kulshrestha et al. [18] propose initializing model weights using a Beta distribution fitted to the normalized input data. The input $d_{i}$ is normalized to [0,1] as in Equation (7)
    (7) $$d_{i}^{\prime }=\frac{d_{i}-\min\left(d\right)}{\max(d)-\min(d)},$$
    and the Beta distribution, as follows in Equation (8),
    (8) $$\mathrm{Beta}\left(\alpha ,\beta |x\right)=\frac{x^{\alpha -1}{\left(1-x\right)}^{\beta -1}}{B(\alpha ,\beta )},$$
    is used to determine shape parameters α and β. Weights are then initialized as W∼Beta(α,β), promoting stable gradient propagation. In related work, small stochastic perturbations may be used to improve exploration of the parameter landscape; however, the present benchmark does not use this mechanism as a hardware-noise model. This approach mitigates the rapid gradient variance decay in large models and improves optimization efficiency, particularly in binary classification tasks [19,20].
  2 **Uniform Norm Initialization**: This strategy leverages a uniform distribution aligned with data-specific statistical properties. Weights *W* are flattened and fitted to a uniform distribution with bounds *a* and *b*, and normalized as in the below Equation (9)
    (9) $$W_{\mathrm{normalized}}=\frac{W-a}{b-a},$$
    to fall within [0,1]. Throughout the training, gradient norms ∥∇W∥ are monitored, with reinitialization or learning rate adjustments if norms drop below $1\times 10^{-5}$, helping maintain stable gradient flow and preventing BPs [21,22].
  3 **Gaussian Initialization**: In Equation (10) parameters are initialized from a Gaussian distribution $N(0,\sigma^{2})$, with σ scaled to the number of layers.
    (10) $$\sigma =\frac{1}{\sqrt{2\times \mathrm{Layers}}}.$$
    This approach is known to improve gradient retention in deep QNNs by minimizing rapid gradient decay. QNNs were implemented with parameterized $R_{X}\left(\theta \right)$ and $R_{Y}\left(\theta \right)$ rotation layers and controlled-Z gates to enhance model expressibility. Gradient norms are monitored, with reinitialization triggered if norms drop below a threshold, helping mitigate BPs [23].
  4 **He Initialization**: The He_normal initialization sets the weights of a layer with $n_{\mathrm{in}}$ input units according to a Gaussian (normal) distribution with mean 0 and variance, which can be defined as in Equation (11)
    (11) $$\sigma^{2}=\frac{2}{n_{\mathrm{in}}}.$$
    Mathematically, the weights can be initialized as follows in Equation (12):
    (12) $$W∼N\left(0,\frac{2}{n_{\mathrm{in}}}\right),$$
    where $n_{\mathrm{in}}$ represents the number of input units in the layer.
    In He_uniform initialization, the weights are sampled from a uniform distribution within the range [−limit,limit], where it can be defined as in Equation (13)
    (13) $$\mathrm{limit}=\sqrt{\frac{6}{n_{\mathrm{in}}}}.$$
    Mathematically, the weights can be initialized as follows in Equation (14)
    (14) $$W∼U\left(-\sqrt{\frac{6}{n_{\mathrm{in}}}},\sqrt{\frac{6}{n_{\mathrm{in}}}}\right),$$
    where $n_{\mathrm{in}}$ denotes the number of input units.
  5 **Xavier Initialization**: The Xavier_normal initialization (Equation (15)) sets the weights of a layer with $n_{\mathrm{in}}$ input units and $n_{\mathrm{out}}$ output units according to a Gaussian (normal) distribution with mean 0 and variance.
    (15) $$\sigma^{2}=\frac{2}{n_{\mathrm{in}}+n_{\mathrm{out}}}.$$
    Mathematically, the weights are initialized as follows in Equation (16)
    (16) $$W∼N\left(0,\frac{2}{n_{\mathrm{in}}+n_{\mathrm{out}}}\right).$$
    In Xavier_uniform initialization, the weights of a layer with $n_{\mathrm{in}}$ input units and $n_{\mathrm{out}}$ output units are sampled from a uniform distribution within the range [−limit,limit], as in Equation (17)
    (17) $$\mathrm{limit}=\sqrt{\frac{6}{n_{\mathrm{in}}+n_{\mathrm{out}}}}.$$
    Mathematically, the weights can be initialized as in Equation (18):
    (18) $$W∼U\left(-\sqrt{\frac{6}{n_{\mathrm{in}}+n_{\mathrm{out}}}},\sqrt{\frac{6}{n_{\mathrm{in}}+n_{\mathrm{out}}}}\right).$$

#### 3.5.1. CNN-Based Initialization

CNN-based initialization uses a small classical convolutional model to generate structured initial parameters for the quantum circuit. The motivation is that a trained or partially trained classical model can provide parameter distributions that are less random than standard initialization and may therefore place the QNN closer to a trainable region of the parameter landscape.

In this benchmark, the CNN branch first extracts a compact feature representation from the input data. The resulting feature vector is passed through a linear projection layer to match the number of trainable QNN parameters. The projected vector is then normalized to the interval required by the quantum rotation parameters and used to initialize the PQC angles. If $h_{\mathrm{CNN}}$ denotes the CNN feature vector and *P* denotes a trainable or fixed projection matrix, the initialized quantum parameter vector is represented as      

(19) $$\begin{array}{c} \theta_{0}=s\cdot \tanh\left(Ph_{\mathrm{CNN}}\right), \end{array}$$

where *s* is a scaling factor chosen so that the initial angles remain within a stable rotation range. After initialization, the QNN parameters are optimized using the same optimizer, learning rate, batch size, and number of epochs as the other mitigation strategies. This ensures that the comparison reflects the effect of initialization rather than differences in the training protocol.

#### 3.5.2. Model-Based Variational Encoder

The model-based mitigation strategy modifies the representation-learning component of the QNN by introducing a variational encoder before measurement. Unlike initialization-only strategies, which change only the starting parameter distribution, the variational encoder changes the way input features and trainable circuit parameters interact. The purpose is to preserve trainability by controlling the expressibility of the circuit and reducing the risk that the parameterized circuit rapidly enters a highly concentrated gradient regime.

Let $x\in R^{n}$ denote the reduced input feature vector used in an *n*-qubit experiment. The variational encoder prepares a parameterized state      

(20) $$\begin{array}{c} |\psi \left(x,\phi \right)\rangle =U_{\mathrm{enc}}\left(x,\phi \right){|0\rangle }^{⊗n}, \end{array}$$

where $U_{\mathrm{enc}}\left(x,\phi \right)$ denotes the encoding unitary and ϕ denotes trainable encoder parameters. The encoded state is then processed by the same two-layer parameterized quantum circuit and Pauli-Z measurement pipeline used for the other methods. This design keeps the training protocol consistent while allowing the benchmark to test whether a structured variational encoder improves gradient retention compared with initialization-only strategies.

#### 3.5.3. Optimization-Based Time-Nonlocal Fourier Parameterization

The optimization-based strategy changes the parameterization of the trainable quantum circuit rather than only changing the initial values. In this approach, trainable circuit angles are expressed through a time-nonlocal Fourier representation. The motivation is that a smoother parameter trajectory can reduce abrupt changes in the optimization landscape and improve gradient stability during training.

A simplified form of the Fourier parameterization is      

(21) $$\begin{array}{c} \theta_{l}\left(t\right)=a_{0}+\sum_{k=1}^{K}\left(a_{k}\cos\left(k\omega t\right)+b_{k}\sin\left(k\omega t\right)\right), \end{array}$$

where $\theta_{l}\left(t\right)$ is the effective circuit parameter at layer or training step *t*, ω is a frequency term, and $\left\{a_{k},b_{k}\right\}$ are trainable Fourier coefficients. The same optimizer, learning rate, batch size, and epoch budget are used so that differences in gradient variance and loss behavior can be attributed to the mitigation strategy rather than to a different training protocol.

### 3.6. Reference Initialization Configuration

A benchmark for barren plateau mitigation benefits from comparison against non-structured stochastic initialization behavior. In the present study, Gaussian initialization and Uniform Norm initialization serve as stochastic reference configurations because they initialize quantum circuit parameters from generic probability distributions rather than from learned features, data-informed projections, or structured variational encoders. These reference settings are trained using the same QNN architecture, optimizer, learning rate, batch size, number of epochs, and qubit configurations as the other methods.

These stochastic reference configurations are used to contextualize whether data-informed or structure-informed methods, such as CNN-based initialization, Beta initialization, the variational encoder, and Fourier parameterization, provide improved gradient retention or convergence behavior under the same experimental conditions. The benchmark therefore compares mitigation behavior relative to both generic stochastic initialization and more structured initialization or parameterization strategies. A fully unmitigated random-initialization control with identical plotting across all figures would further strengthen the benchmark and is identified as an important extension for future work.

### 3.7. Evaluation Metrics

In this project, gradient variance and training loss were chosen as the primary evaluation metrics to assess barren plateaus (BPs) and the performance of quantum neural networks (QNNs). These metrics provide complementary insights into the optimization landscape and the training effectiveness of QNNs. Gradient variance serves as an indicator of BP behavior, but non-zero variance alone does not mathematically prove the absence of barren plateaus. In this benchmark, measurable and non-collapsing variance across the tested qubit range is interpreted as evidence that severe gradient collapse was not observed under the evaluated simulator settings. Training loss is used as a complementary measure of convergence behavior and optimization stability. Below is a detailed explanation of these metrics, their significance, and their role in providing a comprehensive assessment framework.

#### 3.7.1. Gradient Variance

The gradient variance is a robust evaluation metric that quantifies the variability in gradients during the training process. It is particularly useful in identifying and characterizing the phenomenon of barren plateaus (BPs), where gradients diminish to near-zero values over a wide parameter space, impeding effective optimization. Unlike the gradient norm, which evaluates the magnitude of gradients at a single training step, gradient variance provides a statistical measure of how gradients fluctuate across multiple epochs or batches, capturing broader patterns in optimization dynamics.

The gradient variance is mathematically defined as in Equation (22)

(22) $$\mathrm{Var}(∥\nabla_{\theta }C∥)=\frac{1}{N}\sum_{i=1}^{N}{\left(∥\nabla_{\theta }C_{i}∥-\bar{∥\nabla_{\theta }C∥}\right)}^{2}.$$

where:

- $\nabla_{\theta }C_{i}$ represents the gradient vector of the cost function *C* at epoch *i*,
- $∥\nabla_{\theta }C_{i}∥$ is the Euclidean norm (magnitude) of the gradient vector for epoch *i*,
- $\bar{∥\nabla_{\theta }C∥}$ is the mean gradient norm across *N* epochs,
- *N* is the total number of epochs or data points considered.

The gradient vector $\nabla_{\theta }C$ is computed for each epoch, where each component is given by:

$$\frac{\partial C}{\partial \theta_{i}},$$

for i=1,2,…,d, with *d* being the dimensionality of the parameter space.

The Euclidean norm of the gradient is then calculated as follows (Equation (23))

(23) $$∥\nabla_{\theta }C∥=\sqrt{\sum_{i=1}^{d}{\left(\frac{\partial C}{\partial \theta_{i}}\right)}^{2}}.$$

The mean gradient norm over *N* epochs can be determined as follows in Equation (24)

(24) $$\bar{∥\nabla_{\theta }C∥}=\frac{1}{N}\sum_{i=1}^{N}∥\nabla_{\theta }C_{i}∥.$$

Using the computed gradient norms and their mean, the variance can be calculated using the following Equation (25)

(25) $$\mathrm{Var}(∥\nabla_{\theta }C∥)=\frac{1}{N}\sum_{i=1}^{N}{\left(∥\nabla_{\theta }C_{i}∥-\bar{∥\nabla_{\theta }C∥}\right)}^{2}.$$

#### 3.7.2. Training Loss

The training loss quantifies the difference between the predicted and true outputs, reflecting the model’s ability to minimize the cost function during optimization. For supervised learning tasks, it is commonly expressed using the following Equation (26)

(26) $$C=\frac{1}{N}\sum_{i=1}^{N}L\left(y_{i},{\hat{y}}_{i}\right).$$

where

- $L(y_{i},{\hat{y}}_{i})$ is the loss function (e.g., mean squared error, cross-entropy),
- $y_{i}$ and ${\hat{y}}_{i}$ are the true and predicted labels, respectively,
- *N* is the number of data points.

Sudden plateaus in training loss curves indicate regions where gradient magnitudes are too small for meaningful parameter updates, potentially signifying barren plateaus. A smooth and consistent reduction in training loss suggests effective mitigation of BPs, as gradients remain sufficiently large to drive optimization.

### 3.8. Evaluation Scope and Downstream Classification Metrics

The primary objective of this benchmark is to evaluate QNN trainability and barren plateau mitigation behavior. For that reason, the reported analysis focuses on gradient variance and training loss, which directly describe optimization dynamics. Because the datasets used in the benchmark are classification datasets, downstream predictive metrics remain important for assessing whether improved trainability also leads to improved classification performance.

Classification accuracy is commonly defined as      

(27) $$\begin{array}{c} \mathrm{Accuracy}=\frac{N_{\mathrm{correct}}}{N_{\mathrm{total}}}, \end{array}$$

where $N_{\mathrm{correct}}$ is the number of correctly classified samples and $N_{\mathrm{total}}$ is the total number of evaluated samples. For medical image datasets, especially when class imbalance is present, macro-F1, sensitivity, specificity, and area under the receiver operating characteristic curve (AUC) are also relevant. These predictive metrics complement gradient variance and loss by evaluating whether trainability improvements translate into useful classification behavior.

The present benchmark should therefore be interpreted primarily as an optimization and trainability study. Classification-oriented extensions should report accuracy, macro-F1, sensitivity, specificity, and AUC alongside gradient variance and loss, especially for medical image datasets.

Table 1 clarifies the scope of the reported evaluation. Gradient variance and training loss are the reported metrics because the study benchmarks barren plateau mitigation and QNN trainability. Predictive metrics such as accuracy, macro-F1, sensitivity, specificity, and AUC are not reported in the current benchmark; they are therefore identified as necessary additions for future classification-oriented evaluations of the same mitigation strategies.

## 4. Results and Discussion

### 4.1. Variance Analysis: Mitigation of Barren Plateaus

**Figure 1:**
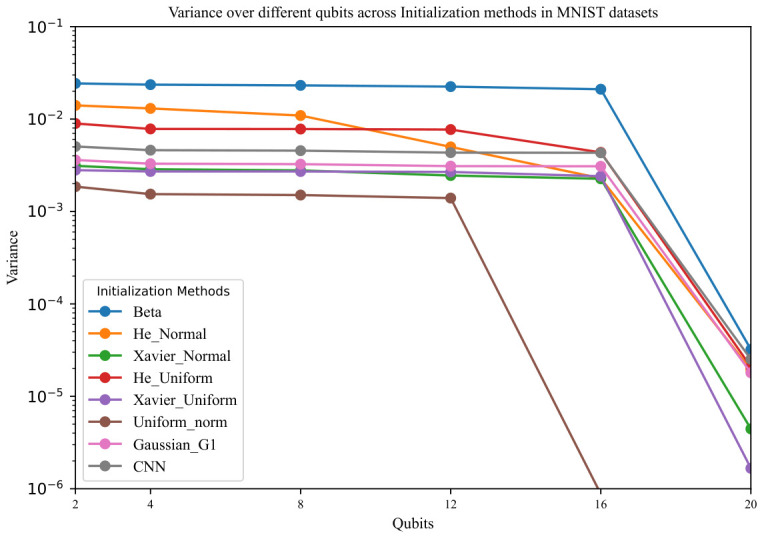
Variance Analysis Across Qubits for different Initialization Methods on MNIST Dataset.

**Figure 2:**
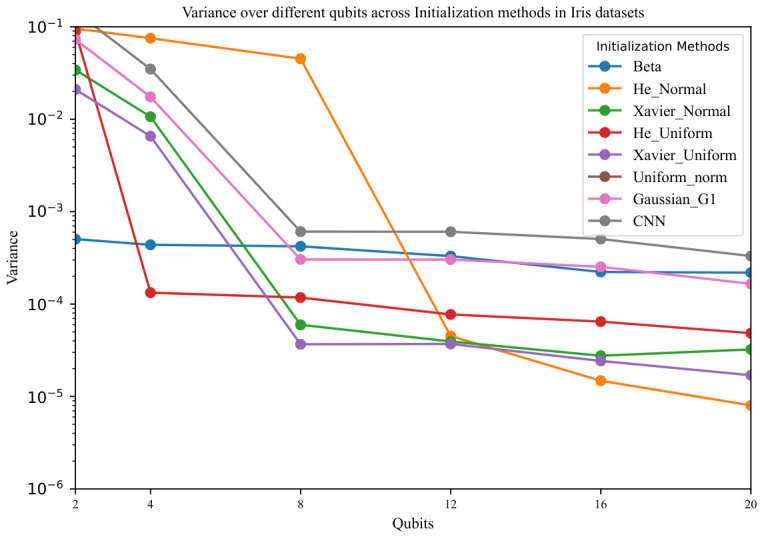
Variance Analysis Across Qubits for different Initialization Methods on Iris Dataset.

**Figure 3:**
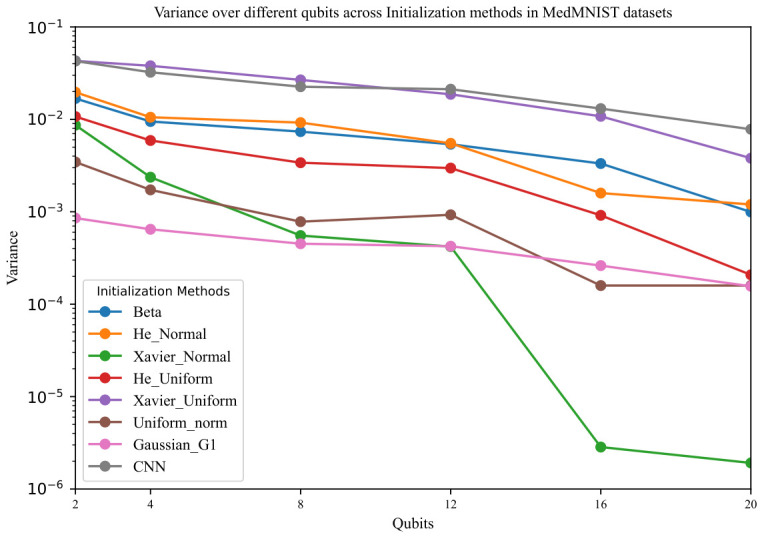
Variance Analysis Across Qubits for different Initialization Methods on MedMNIST Dataset.

Figure 1, Figure 2 and Figure 3 illustrate variance trends across the eight initialization methods. Among them CNN emerges as the most effective, consistently maintaining the highest variance levels across datasets and qubit configurations. For instance, in Figure 2 (Iris dataset), CNN achieves a variance of approximately $10^{-3}$ for most qubit settings, demonstrating strong BP mitigation among all the methods. Beta initialization also performs well, maintaining variances around $10^{-1}$ to $10^{-3}$ across all configurations, with minimal decay even at higher qubits like 16 and 20. These results indicate that CNN-based and Beta initialization provide comparatively strong gradient-retention behavior under the tested benchmark conditions.

**Figure 4:**
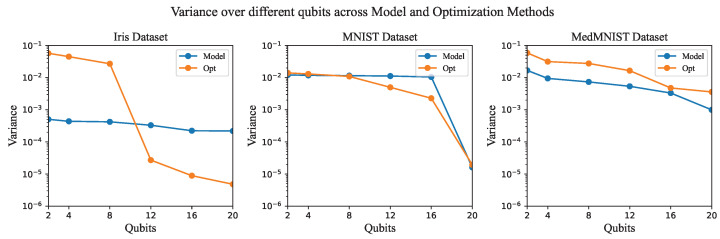
Variance Analysis Across Qubits for Model and Optimization Methods on MNIST, Iris and MedMNIST Datasets.

Figure 4 compares the model-based and optimization-based methods across the three datasets. The model-based method generally maintains variance around $10^{-3}$ to $10^{-2}$ in several higher-qubit configurations, indicating stable trainability in those settings. The optimization-based method shows stronger variance retention in some smaller-qubit configurations, but its behavior varies by dataset and qubit count. For example, in the Iris setting, the optimization-based method drops below $10^{-5}$ in some configurations, whereas the model-based method retains approximately $10^{-3}$ even at 20 qubits. These trends indicate that the relative advantage of model-based and optimization-based mitigation depends on dataset complexity and circuit size.

Several methods show variance decay as the number of qubits increases, particularly for higher qubit configurations such as 16 and 20 qubits. This behavior is consistent with the expected difficulty of training larger variational circuits. However, the variance curves remain measurable under the tested shallow-circuit simulator settings, suggesting that severe gradient collapse was not observed in these configurations. He-normal and He-uniform show moderate variance retention, while Uniform Norm and Xavier-based methods provide comparatively stable behavior in several intermediate qubit settings. These results support the use of the benchmark for comparing relative trainability trends, rather than for claiming universal BP-free behavior. Figure 4 represents the variance trends for optimization and model-based methods across the three datasets. Model-based strategies outperform optimization-based methods in higher qubit settings in the maximum cases, maintaining variance around $10^{-3}$ to $10^{-2}$ across most configurations. Optimization-based methods consistently outperform model-based methods in variance retention across all datasets and smaller qubit configurations (Figure 4); the model-based method retains a variance of $10^{-3}$ even for 20 qubits, while the optimization-based method drops below $10^{-5}$ for Iris datasets.

These results demonstrate the usefulness of the benchmark for comparing BP mitigation behavior under controlled simulator settings. Across the tested qubit configurations and datasets, the evaluated methods generally maintained measurable gradient variance, suggesting that severe gradient collapse was not observed in the shallow-circuit regime considered here. The results should be interpreted as empirical trainability evidence for the tested configurations rather than as a universal guarantee of BP elimination in deeper circuits or noisy quantum hardware.

### 4.2. Loss Curve Analysis: Evaluating Convergence and Stability

**Figure 5:**
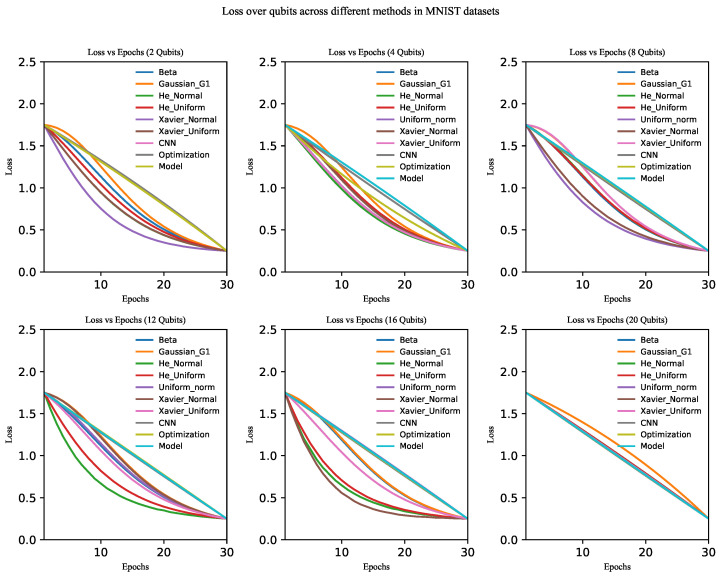
Loss Analysis Across Qubits for different Methods on MNIST Dataset.

**Figure 6:**
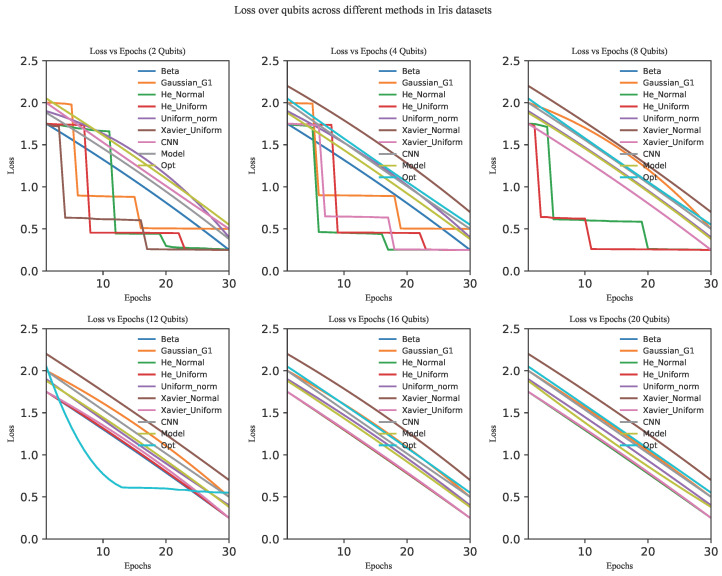
Loss Analysis Across Qubits for different Methods on Iris Dataset.

**Figure 7:**
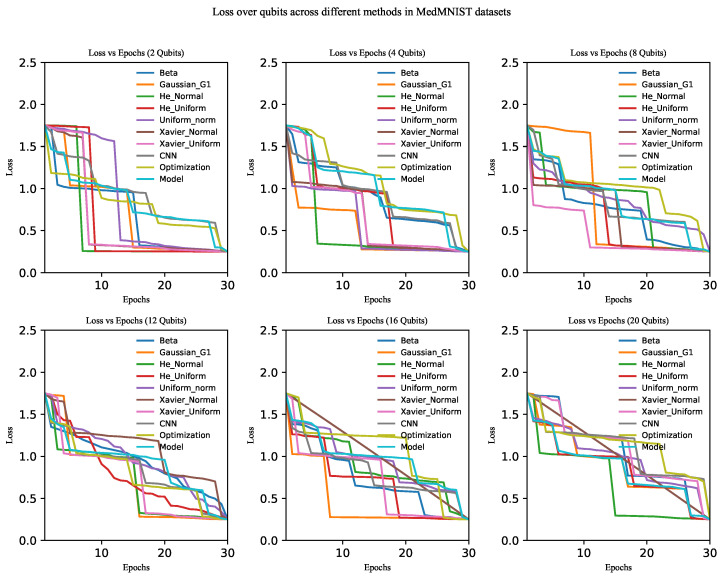
Loss Analysis Across Qubits for different Methods on MedMNIST Dataset.

To complement the gradient-variance analysis, we evaluate training-loss curves across MNIST, Iris, and MedMNIST datasets, as shown in Figure 5, Figure 6 and Figure 7. Loss curves provide information about convergence speed and optimization stability for the 10 evaluated methods over 30 epochs and six qubit configurations. While loss reduction does not by itself prove the absence of barren plateaus, stable loss convergence together with measurable gradient variance provides stronger evidence that the evaluated QNNs remained trainable under the tested simulator settings. Figure 5 illustrates the loss trends for MNIST. CNN-based initialization shows the fastest convergence and the lowest final loss in several qubit settings, reducing the loss to approximately 0.1 for the 16-qubit configuration. The model-based and optimization-based methods also show stable convergence, with representative final losses around ∼0.15 and ∼0.2, respectively. Gaussian initialization is comparatively weaker in higher-qubit configurations, with final losses exceeding 0.6 in some cases, while Uniform Norm and Xavier-normal show moderate convergence behavior with losses around ∼0.3 for several configurations.

For the Iris dataset, shown in Figure 6, the optimization-based method converges rapidly in smaller-qubit configurations, reaching near-zero loss within approximately 10 epochs for the 2-qubit setting. CNN-based and Beta initialization show stable convergence across multiple configurations, with CNN-based initialization reaching final losses below 0.2 in several settings. Gaussian initialization again shows weaker convergence, while Xavier-based methods exhibit slower but relatively stable optimization behavior.

For MedMNIST, CNN-based initialization shows strong convergence behavior, with loss values decreasing below approximately 0.2 across the tested configurations in Figure 7. Beta and model-based methods also show stable behavior in several settings, particularly around 8 and 12 qubits, with final losses below approximately 0.3. Uniform Norm and Xavier-normal show moderate convergence, with losses around ∼0.4 in several configurations. Gaussian initialization is comparatively weaker, with losses exceeding 0.6 in some larger configurations and early oscillations in smaller settings. Overall, the loss curves suggest that the evaluated QNNs remained trainable under the tested simulator conditions, although the results should not be interpreted as a general proof of scalability or BP elimination in deeper or noisy hardware settings.

### 4.3. Benchmark Viewpoints

The joint analysis of gradient variance and training loss provides a structured view of BP mitigation behavior. Gradient variance captures whether gradients remain measurable across qubit settings, while training loss indicates whether the optimizer can still make progress during learning. These two metrics are complementary: a method may preserve gradient variance but converge slowly, or it may reduce loss rapidly in smaller settings while showing weaker variance retention at larger qubit counts.

Within the tested shallow-circuit simulator setting, CNN-based initialization and Beta initialization showed strong empirical behavior in both variance retention and loss convergence. Model-based and optimization-based strategies also provided useful trainability behavior, although their performance varied across datasets and qubit settings. Gaussian initialization was comparatively weaker in higher-dimensional cases but still maintained measurable variance under the evaluated configurations.

The benchmark therefore provides a controlled comparison of mitigation behavior rather than a universal ranking of methods. The relative usefulness of each strategy may change with circuit depth, encoding method, hardware noise, optimizer choice, and dataset structure.

### 4.4. Circuit Depth and Scalability Considerations

The benchmark uses a two-layer parameterized quantum circuit to maintain computational feasibility across six qubit settings, three datasets, and 10 mitigation strategies. This design enables controlled comparison under a shared experimental protocol, but it does not fully characterize barren plateau behavior in deeper ansatzes. Since barren plateaus are known to become more severe as circuit depth, expressibility, and system size increase, the conclusions should be interpreted within the shallow-circuit regime evaluated in this study.

Deeper circuits with 5, 10, or 20 parameterized layers would provide a stronger stress test of mitigation behavior. Such experiments are particularly important for determining whether the observed gradient retention persists as circuit depth increases. Therefore, the present benchmark provides a controlled first comparison across qubit settings and datasets, while deeper-circuit benchmarking remains an important direction for extending the evaluation.

### 4.5. Practical QNN Setbacks and Benchmark Implications

The benchmark should be interpreted in the context of several practical QNN limitations. First, gradient concentration becomes more severe as the number of qubits and circuit depth increase. This is the central barren plateau issue examined in the study, although the reported conclusions are limited to the tested qubit range and two-layer circuit design.

Second, decoherence and imperfect gate fidelities can affect both circuit depth and classification accuracy on physical quantum devices. The present experiments use simulator backends without explicit hardware-noise models; therefore, the results do not establish robustness to decoherence, gate errors, or readout noise. Hardware-aware extensions should evaluate the same mitigation strategies under depolarizing noise, amplitude damping, phase damping, readout error, and physical QPU execution.

Third, mapping high-dimensional classical data into quantum states remains a central bottleneck for image-based QNNs. MNIST and MedMNIST images contain many more raw features than can be directly represented by the tested qubit configurations. Dimensionality reduction, feature selection, and rotation-based encoding are therefore necessary preprocessing steps, and these choices can influence both gradient behavior and downstream classification quality.

Fourth, QNN benchmarking imposes a heavy computational load because training may require repeated circuit evaluations for each batch, parameter update, qubit setting, and mitigation strategy. This cost increases with qubit number, circuit depth, number of shots, and the number of compared methods. The simulator-based two-layer design used in this benchmark controls this cost while enabling broad comparison, but larger-scale studies will require more efficient batching, parallel simulation, hardware-aware execution, and deeper-circuit analysis.

### 4.6. Discussion

The results indicate that the evaluated mitigation strategies can preserve trainability under the tested simulator conditions. In particular, measurable gradient variance and stable loss reduction were observed across Iris, MNIST, and MedMNIST for the selected qubit settings. CNN-based initialization and Beta initialization showed especially strong behavior in this benchmark, suggesting that structured or data-informed initialization can help place the QNN parameters in more trainable regions of the optimization landscape.

At the same time, the findings should be interpreted carefully. The benchmark uses a two-layer circuit, simulator backends, and controlled preprocessing. These choices make broad comparison feasible, but they do not fully reproduce the conditions under which barren plateaus may become more severe, such as deeper ansatzes, noisy physical hardware, larger numbers of trainable parameters, or more demanding image-encoding schemes. Therefore, the benchmark supports conclusions about relative trainability under the evaluated conditions, not a general proof that the tested methods eliminate barren plateaus in all QNN settings.

The results also highlight the importance of reporting both optimization-oriented and task-oriented metrics. Gradient variance and loss curves are appropriate for studying trainability, while classification metrics such as accuracy, macro-F1, sensitivity, specificity, and AUC are needed to determine whether improved trainability translates into predictive performance. Future benchmark extensions should report both types of metrics.

## 5. Related Work

Barren plateaus (BPs) are a significant challenge in training parameterized quantum circuits (PQCs) and quantum neural networks (QNNs), particularly as the number of qubits and circuit depth increase. The phenomenon, first identified by McClean et al. [24], describes regions in parameter space where gradients vanish exponentially, hindering effective optimization in variational quantum algorithms (VQAs) and PQCs. Recent research has explored various strategies to mitigate BPs through initialization techniques, model-based approaches, and optimization strategies.

### 5.1. Initialization-Based Mitigation Strategies

Initialization strategies are fundamental to ensuring effective gradient flow during training and preventing barren plateaus. Grant et al. proposed identity block initialization, which strategically sets parameters to preserve gradients in deep circuits [15]. Sauvage et al. introduced FLIP, an adaptable initialization method for PQCs of varying sizes [25]. Sack et al. leveraged classical shadow protocols to optimize initial parameters, reducing gradient decay in highly entangled circuits [26]. Bayesian learning-based initialization, as proposed by Rad et al., predicts optimal parameter configurations using Bayesian priors, improving training efficiency [27]. Kulshrestha and Safro developed BeInit, a beta distribution-based initialization method that ensures uniform gradient retention across circuit layers [18]. Zhang et al. employed depth-scaled Gaussian distributions to counter gradient decay in deep quantum models [22]. Friedrich and Maziero utilized pre-trained classical neural networks to initialize QNN parameters, bridging classical and quantum optimization [28]. Mele et al. proposed a transferability framework for initializing parameters from simpler quantum systems to avoid flat optimization landscapes [29]. Grimsley et al. introduced an adaptive variational quantum eigensolver (VQE) to dynamically refine initial parameters for complex systems [30]. Liu et al. utilized transfer learning-inspired initialization, transferring parameter distributions from small-scale tasks to enhance scalability in larger circuits [31]. Park and Killoran proposed a Hamiltonian variational ansatz optimized for initialization, eliminating barren plateaus by ensuring gradient retention [32].

Friedrich et al. introduced Classical CNN-based Initialization, utilizing a pre-trained classical neural network to generate starting parameters for QNNs. This approach demonstrated success in mitigating BPs by leveraging well-distributed parameter values from classical models, enhancing stability in gradient-based optimization [28]. Collectively, these studies underscore the importance of carefully chosen parameter distributions, with recent work suggesting that strategies like Beta and Gaussian Initializations outperform traditional uniform distributions in BP-prone landscapes.

### 5.2. Model-Based Mitigation Strategies

Model-based approaches focus on designing robust quantum circuit architectures that balance expressibility and gradient flow. Li et al. introduced Variational Shadow Quantum Learning (VSQL), which leverages classical shadows for efficient classification [33]. Bharti and Haug developed a quantum-assisted simulator that avoids classical-quantum feedback, mitigating barren plateaus effectively [34]. Du et al. proposed Quantum Circuit Architecture Search (QCAS), dynamically generating ansatz structures tailored to specific tasks to prevent BPs [35]. Zhang et al.’s Quark framework eliminates gradient computation, avoiding BPs entirely [36]. Selvarajan et al. employed variational encoders for dimensionality reduction to avoid over-entanglement, ensuring effective gradient flow [37]. Tuysuz et al. introduced a classical splitting ansatz to enhance scalability and gradient retention [38]. Kashif and Al-Kuwari’s ResQNets architecture incorporated residual connections to address vanishing gradients in high-dimensional quantum circuits [39]. Shin et al. proposed Layerwise Quantum Convolutional Neural Networks (LQCNNs) to mitigate barren plateaus in circuits with large qubit states [40]. Zhang et al. demonstrated the inherent resistance of finite local-depth circuits with long-range entanglement to barren plateaus, even in complex optimization tasks [41]. Additionally, Marrero et al. investigated how controlled expressibility within quantum circuits can reduce BP occurrence by avoiding overly complex ansatz structures [17].

### 5.3. Optimization-Based Mitigation Strategies

Optimization-based strategies refine training processes to effectively address BPs. Ostaszewski et al. optimized PQC structures to retain gradients during training, enhancing trainability [42]. Skolik et al. introduced layerwise learning, incrementally training circuits to minimize gradient decay in deeper networks [43]. Gharibyan et al. proposed hierarchical learning, dynamically scaling training processes for large variational circuits to reduce the risk of BPs [44]. Haug and Kim developed an adaptive learning rate approach to maintain gradient magnitudes, preventing optimization stagnation [45]. Mele et al. employed noise-induced shallow circuits to stabilize gradients, demonstrating improved trainability in large systems [46]. Sannia et al. introduced engineered dissipation processes to counteract BPs during training by injecting controlled noise [47]. Zambrano et al. optimized geometric entanglement to preserve gradient effectiveness in quantum systems [48]. Broers and Mathey introduced time-nonlocal optimization, leveraging Fourier-based parameterization to mitigate barren plateaus [49]. Heyraud et al. proposed efficient trainability estimation tools to guide optimization processes in large quantum systems [50]. Kieferova et al. applied Rényi divergence in generative quantum training, showcasing its utility in avoiding gradient vanishing during optimization [51]. Sciorilli et al. explored qubit-efficient encoding for optimization, demonstrating reduced BP risks in parameterized circuits [52]. Falla et al. investigated graph embedding techniques for optimizing parameter transferability, highlighting their effectiveness in mitigating barren plateaus during large-scale quantum optimization [53].

### 5.4. Evaluation Tools and Theoretical Insights

Evaluation tools and theoretical insights are critical for understanding barren plateaus and guiding mitigation strategies. Patti et al. developed an entanglement-based framework using gradient metrics to assess BP risks, providing a structured evaluation framework [54]. Larocca et al. introduced diagnostic tools derived from quantum optimal control, identifying BPs and optimizing cost functions to enhance trainability [55]. Park and Kim proposed a software testing framework for QNNs, integrating runtime analysis and optimization to detect and resolve BPs [56]. Kashif et al. analyzed advanced initialization strategies, employing comprehensive evaluation metrics to study their effectiveness in alleviating BPs [21]. Additionally, Cerezo et al. provided foundational insights into the connection between cost function locality and BP emergence, establishing that local cost functions are less likely to exhibit BPs compared to global cost functions. This work highlighted that selecting appropriate cost functions can be as crucial as initialization and architecture adjustments for BP mitigation [57]. The landscape of BP mitigation in quantum machine learning has advanced significantly, with effective strategies emerging from diverse areas such as initialization techniques, architecture design, and optimized training processes. This project builds on these foundational works by benchmarking multiple BP mitigation strategies across standard datasets, systematically evaluating initialization, model-based, and optimization techniques to identify optimal configurations for scalable QNNs. The findings from this study contribute to the growing body of research focused on making QNNs more trainable and scalable, addressing a critical bottleneck in quantum machine learning.

## 6. Conclusions

This study presented a comparative benchmark of barren plateau mitigation strategies for quantum neural networks across standard and medical image datasets. Ten mitigation strategies were evaluated across six qubit settings using a shared simulator-based QNN setup. The results show that, under the tested two-layer circuit configurations, the evaluated strategies generally maintained measurable gradient variance and stable loss reduction, suggesting that severe barren plateau behavior was not observed in this controlled benchmark.

CNN-based initialization and Beta initialization showed particularly strong empirical behavior in gradient retention and convergence speed, while Gaussian initialization was comparatively weaker in higher-dimensional settings. However, the findings should not be interpreted as proof that these methods eliminate barren plateaus in general. Deeper circuits, explicit noise models, physical quantum hardware, and broader medical image datasets may produce different trainability behavior.

The main contribution of the study is therefore a reproducible benchmark structure and comparative trainability analysis rather than a claim of universal BP elimination. Future work should extend the benchmark to deeper ansatzes, explicit hardware-noise models, real QPU execution, additional medical image datasets, and standard classification metrics such as accuracy, macro-F1, sensitivity, specificity, and AUC.

### Limitations and Future Scope

Despite the usefulness of the benchmark, several limitations should be noted. First, the experiments use Iris, MNIST, and MedMNIST as representative datasets. Although these datasets provide increasing levels of complexity, they do not cover the full diversity of medical imaging tasks or large-scale quantum machine learning applications. Future work should evaluate additional medical image datasets and compare multiple MedMNISTv2 subsets when dataset-specific conclusions are required.

Second, the QNN architecture uses a two-layer parameterized circuit. This setting enables comparison across many methods and qubit configurations, but it does not fully test the deeper-circuit regimes in which barren plateaus may become more severe. Future work should include deeper circuits, such as 5-, 10-, and 20-layer ansatzes, to evaluate how mitigation behavior changes with circuit depth and expressibility.

Third, the experiments are simulator-based and do not explicitly include hardware-noise models. As a result, the benchmark does not establish robustness to decoherence, imperfect gate fidelities, readout noise, or calibration drift on physical quantum devices. Hardware-aware studies should evaluate the same mitigation strategies under realistic noise models and on real QPUs.

Fourth, the present evaluation focuses on gradient variance and training loss. These metrics are appropriate for analyzing trainability and barren plateau behavior, but they do not fully characterize downstream classification performance. Future benchmark extensions should report accuracy, macro-F1, sensitivity, specificity, and AUC, particularly for medical image datasets where class imbalance and diagnostic reliability are important.

Finally, the preprocessing and encoding of high-dimensional image data remain important open issues. Mapping image features into a small number of qubits requires dimensionality reduction or feature selection, and different encoding choices may affect both gradient behavior and classification performance. Future work should compare rotation-based, amplitude-based, and patch-based encodings under shared benchmark conditions.

## Figures and Tables

**Table 1:** Evaluation scope of the benchmark and downstream classification metrics relevant for future extensions.

Method Group	Gradient Variance	Training Loss	Accuracy	Macro-F1/AUC
Stochastic reference initialization	Reported	Reported	Not reported	Not reported
Beta initialization	Reported	Reported	Not reported	Not reported
Gaussian initialization	Reported	Reported	Not reported	Not reported
Uniform Norm initialization	Reported	Reported	Not reported	Not reported
CNN-based initialization	Reported	Reported	Not reported	Not reported
He-normal/He-uniform	Reported	Reported	Not reported	Not reported
Xavier-normal/Xavier-uniform	Reported	Reported	Not reported	Not reported
Model-based variational encoder	Reported	Reported	Not reported	Not reported
Optimization-based Fourier parameterization	Reported	Reported	Not reported	Not reported

## Data Availability

The data presented in this study are available on request from the corresponding author.
